# Effective injury forecasting in soccer with GPS training data and machine learning

**DOI:** 10.1371/journal.pone.0201264

**Published:** 2018-07-25

**Authors:** Alessio Rossi, Luca Pappalardo, Paolo Cintia, F. Marcello Iaia, Javier Fernàndez, Daniel Medina

**Affiliations:** 1 Department of Computer Science, University of Pisa, Pisa, Italy; 2 ISTI, National Research Council, Pisa, Italy; 3 Department of Biomedical Science for Health, University of Milan, Milan, Italy; 4 Sports Science and Health Department, FC Barcelona, Barcelona Spain; 5 Athletic Care Department, Philadelphia 76ers, Philadelphia, Pennsylvania, United States of America; Universidade de Tras-os-Montes e Alto Douro, PORTUGAL

## Abstract

Injuries have a great impact on professional soccer, due to their large influence on team performance and the considerable costs of rehabilitation for players. Existing studies in the literature provide just a preliminary understanding of which factors mostly affect injury risk, while an evaluation of the potential of statistical models in forecasting injuries is still missing. In this paper, we propose a multi-dimensional approach to injury forecasting in professional soccer that is based on GPS measurements and machine learning. By using GPS tracking technology, we collect data describing the training workload of players in a professional soccer club during a season. We then construct an injury forecaster and show that it is both accurate and interpretable by providing a set of case studies of interest to soccer practitioners. Our approach opens a novel perspective on injury prevention, providing a set of simple and practical rules for evaluating and interpreting the complex relations between injury risk and training performance in professional soccer.

## Introduction

Injuries of professional athletes have a great impact on the sports industry, due to their influence on the mental state of the individuals and the performance of a team [1, 2]. Furthermore, the cost associated with a player’s recovery and rehabilitation is often considerable, both in terms of medical care and missed earnings deriving from the popularity of the player himself [3]. Recent research demonstrates that injuries in Spain cause about 16% of season absence by professional soccer players, corresponding to a cost of around 188 million euros per season [4]. It is not surprising, hence, that injury forecasting is attracting a growing interest from researchers, managers, and coaches, who are interested in intervening with appropriate actions to reduce the likelihood of injuries of their players.

Historically, academic work on injury forecasting has been deterred by the limited availability of data describing the physical activity of players. Nowadays, the Internet of Things have the potential to change rapidly this scenario thanks to Electronic Performance and Tracking Systems (EPTS), new tracking technologies that provide high-fidelity data streams extracted from every training and game session [5, 6]. These data depict in detail the movements of players on the playing field [5, 6] and have been used for many purposes, from identifying training patterns [7] to automatic tactical analysis [5, 8, 9]. Despite this wealth of data, little effort has been put on investigating injury forecasting in professional soccer so far [10, 11, 12]. State-of-the-art approaches provide just a preliminary understanding of which variables affect the injury risk, while an evaluation of the potential of statistical models to forecast injuries is still poor. A major limit of existing studies is that they are mono-dimensional, i.e., they use just one variable at a time to estimate injury risk, without fully exploiting the complex patterns underlying the available data.

Professional soccer clubs are interested in practical, usable and interpretable models as a decision making support for coaches and athletic trainers [13]. In this perspective the creation of injury forecasting models poses many challenges. On one hand, injury forecasters must be highly accurate, as models which frequently produce “false alarms” are useless. On the other hand, a “black box” approach (e.g., a deep neural network) is not desirable for practical use since it does not provide any insights about the reason behind the injuries. It goes hence without saying that injury forecasting models must achieve a good tradeoff between accuracy and interpretability.

In this paper, we consider injury prediction as the problem of forecasting that a player will get injured in the next training session or official game, given his recent training workload. We observe that existing mono-dimensional approaches are not effective in practice due to their low precision (< 5%), and we propose a multi-dimensional, easy-to-interpret and fully data-driven approach which forecasts injuries with a better precision (50%); we validate this result by simulating the usage of our forecaster over a season, with new training data available as the season goes by. Our approach is entirely based on automatic data collection through standard GPS sensing technologies and can be a valid supporting tool to the decision making of a soccer club’s staff. This is crucial since the decisions of managers and coaches, and hence the success of soccer clubs, also depend on what they measure, how good their measurements are, the quality of predictions and how well these predictions are understood.

### Related work

The relationship between training workload and injury risk has been widely studied in the sports science literature [14, 15, 16, 17, 18]. For example Gabbett et al. [14, 15, 17, 19] investigate the case of rugby and find that a player has a high injury risk when his workloads are increased above certain thresholds. To assess injury risk in cricket, Hulin et al. [20] propose the Acute Chronic Workload Ratio (ACWR), i.e., the ratio between a player’s acute workload and his chronic workload. When the acute workload is lower than the chronic workload, cricket players are associated with a low injury risk. In contrast, when the acute/chronic ratio is higher than 2, players have an injury risk from 2 to 4 times higher than the other group of players. Hulin et al. [20] and Ehrmann et al. [11] find that injured players, in both rugby and soccer, show significantly higher physical activity in the week preceding the injury with respect to their seasonal averages.

In skating, Foster et al. [21] measure training workload by the session load, i.e., the product of the perceived exertion and the duration of the training session. When the session load outweighs a skater’s ability to fully recover before the next session, the skater suffers from the so-called “overtraining syndrome”, a condition that can cause injury [21]. In basketball, Anderson et al. [18] find a strong correlation between injury risk and the so-called monotony, i.e., the ratio between the mean and the standard deviation of the session load recorded in the past 7 days. Moreover, Brink et al. [8] observe that injured young soccer players (age < 18) recorded higher values of monotony in the week preceding the injury than non-injured players.

Venturelli et al. [12] perform several periodic physical tests on young soccer players (age < 18) and find that jump height, body size and the presence of previous injuries are significantly correlated with the probability of thigh strain injury. Talukder et al. [22] create a classifier to predict 19% of the injuries that occurred in NBA. They also show that the most important features for predicting injuries are the average speed, the number of past competitions played, the average distance covered, the number of minutes played to date and the average field goals attempted. An attempt to injury forecasting in soccer has been made by Kampakis [23], although it considers a reduced set of features obtaining an accuracy that is, in the best scenario, not significantly better than random classifiers.

## Material and method

### Data collection and feature extraction

We set up a study on twenty-six Italian professional male players (age = 26±4 years; height = 179±5 cm; body mass = 78±8 kg) during season 2013/2014. Six central backs, three fullbacks, seven midfielders, eight wingers and two forwards were recruited. Participants gave their written informed consent to participate in the study.

We monitored the physical activity of players during 23 weeks–from January 1st to May 31st, 2014 –using portable 10 Hz GPS devices integrated with a 100Hz 3-D accelerometer, a 3D gyroscope, a 3D digital compass (STATSports Viper). The devices were placed between the players’ scapulae through a tight vest. We recorded a total of 931 individual training sessions during the 23 weeks. From the data collected by the devices, we extracted a set of training workload indicators through the software package Viper Version 2.1 provided by STATSports 2014.

The club’s medical staff recorded 23 non-contact injuries during the study. According to the UEFA regulations [24], a non-contact injury is defined as any tissue damage sustained by a player that causes absence in physical activities for at least the day after the day of the onset. We observed that 19 out of 23 injuries are associated with players who got injured at least once in the past. In particular, half of the players never get injured during the study, while the others get injured once (seven players), twice (five players) or four times (one player). For every player, we collected information about age, body mass index, height and role on the field. Moreover, for every single training session of a player, we collected information about the play time in the official game before the training session and the number of official games played before the training session.

From the players’ GPS data we extract 12 features describing different aspects of the workload in a training session [25]. Two features–Total Distance (d_TOT_) and High Speed Running Distance (d_HSR_)–are kinematic, i.e., they quantify a player’s overall movement during a training session. Three features–Metabolic Distance (d_MET_), High Metabolic Load Distance (d_HML_) and High Metabolic Load Distance per minute (d_HML/m_)–are metabolic, i.e., they quantify the energy expenditure of a player’s overall movement during a training session. The remaining seven features–Explosive Distance (d_EXP_), number of accelerations above 2m/s2 (Acc_2_), number of accelerations above 3m/s2 (Acc_3_), number of decelerations above 2m/s2 (Dec_2_), number of decelerations above 3m/s2 (Dec_3_), Dynamic Stress Load (DSL) and Fatigue Index (FI)–are mechanical features describing a player’s overall muscular-scheletrical load during a training session. In addition, we associated a player’s training session with feature PI, indicating the number of the player’s previous injuries up to that session. Table 1 and S1 Appendix provides the description and some statistics of the workload features extracted from the GPS data, respectively.

**Table 1:** Training workload features used in our study. Description of the training workload features extracted from GPS data and the players’ personal features collected during the study. We defined four categories of features: kinematic features (blue), metabolic features (red), mechanical features (green) and personal features (white).

d_TOT_	Distance in meters covered during the training session
d_HSR_	Distance in meters covered above 5.5m/s
d_MET_	Distance in meters covered at metabolic power
d_HML_	Distance in meters covered by a player with a Metabolic Power is above 25.5W/Kg
d_HML/m_	Distance in meters covered by a player with a Metabolic Power is above 25.5W/Kg per minute
d_EXP_	Distance in meters covered above 25.5W/Kg and below 19.8Km/h
Acc_2_	Number of accelerations above 2m/s2
Acc_3_	Number of accelerations above 3m/s2
Dec_2_	Number of decelerations above 2m/s2
Dec_3_	Number of decelerations above 3m/s2
DSL	Total of the weighted impacts of magnitude above 2g. Impacts are collisions and step impacts during running
FI	Ratio between DSL and speed intensity
Age	age of players
BMI	Body Mass Index: ratio between weight (in kg) and the square of height (in meters)
Role	Role of the player
PI	Number of injuries of the players before each training session
Play time	Minutes of play in previous games
Games	Number of games played before each training session

### Multi-dimensional and data-driven injury forecaster

We construct a multi-dimensional model to forecast whether or not a player will get injured based on his recent training workload. The construction of the injury forecaster consists of two phases. In the first phase (training dataset construction), given a set of features *S*, a training dataset *T* is created where each example refers to a single player’s training session and consists of: (i) a vector of features describing both the player’s personal features and his recent workload, including the current training session; (ii) an injury label, indicating whether (1) or not (0) the player gets injured in the next game or training session. In the second phase (model construction and validation), a decision tree learner is used to train an injury classifier on the training dataset *T*.

#### Phase 1: Training dataset construction

From the features extracted from GPS data, which are described in Table 1, we construct a training dataset *T* consisting of 55 features and 952 examples (i.e., individual training sessions). S4 Appendix provides an example of the construction of *T*. These 55 features are:

- ***18 daily features*:** the 12 workload features extracted from the GPS data and the 6 personal features described in Table 1.
- ***12* EWMA features:** 12 features computed as the Exponential Weighted Moving Average (EWMA) of the 12 workload features in Table 1. The EWMA decreases exponentially the weights of the values according to their recency, i.e., the more recent a value, the more it is weighted in an exponential function according to a decay α = 2/(span+1). In our experiments we consider a span equal to six (see S5 Appendix).
- ***12* ACWR *features*:** 12 features consisting of the ACWR of the 12 workload features in Table 1. Given a feature, the ACWR of a player is the ratio between (i) the player’s acute workload, computed as the average of the values of the feature in the last 6 days; (ii) the player’s chronic workload, computed as the average of the values of the feature in the last 27 days [26].
- ***12* MSWR *features*:** 12 features consisting of the monotony of the 12 workload features in Table 1. Given a feature, the monotony of a player is the ratio between the mean and the standard deviation of the values of the feature in the last week [3, 10, 18].
- ***1 previous injury feature*:** to take into account both the number of a player’s previous injuries and their distance to the current training session we compute feature PI^(WF)^, the EWMA of feature PI computed with a span equal to six. PI^(WF)^ reflects the distance between the current training session and the training session when the player returned to regular training after an injury. PI^(WF)^ = 0 indicates that the player never got injured in the past; PI^(WF)^ > 0 indicates that he got injured at least once in the past; PI^(EWMA)^ > 1 indicate that he got injured more than once in the past (see S6 Appendix).

**Fig 1:**
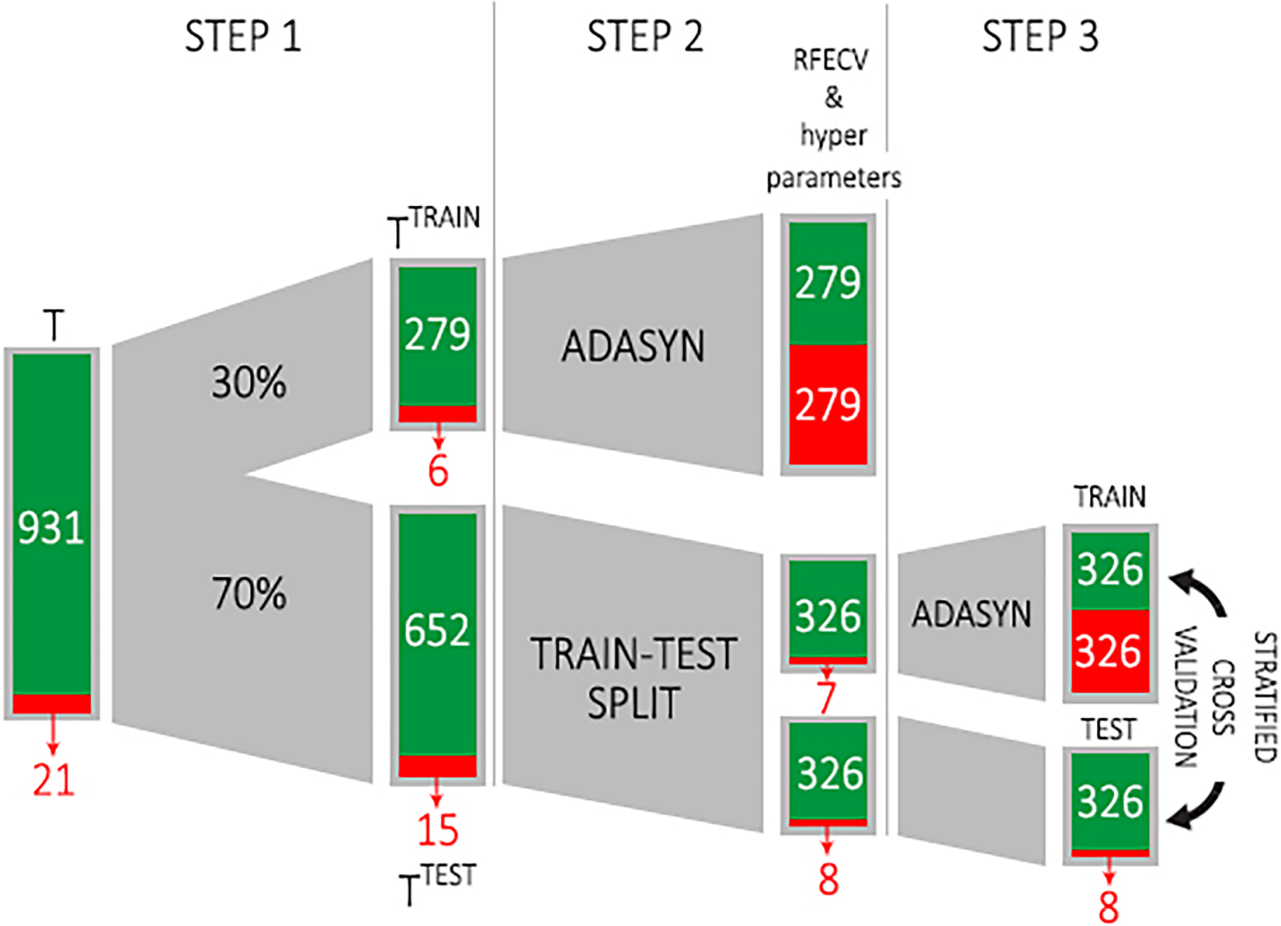
Construction of the training dataset and the forecasting model. In step 1 we split the dataset into two parts: T^TRAIN^ (30% of T) and T^TEST^ (70% of T). We then oversample the minority class in T^TRAIN^ by using ADASYN, select the most important features and fit the hyper parameters (Step 2). We then split T^TEST^ into two folds in order to perform a stratified cross validation (step 3).

We select 30% of *T* and obtain T^TRAIN^ (step 1 and 2 in Fig 1) to perform a feature selection process to determine the most relevant features for classification using Recursive Feature Elimination with Cross-Validation (RFECV; we use the publicly available Python package scikit-learn to perform RFECV and to train and validate the decision tree– http://scikit-learn.org/) [27]. In RFECV, the subset of features producing the maximum score on the validation data is considered to be the best feature subset [27]. The feature selection process is aimed at reducing the dimensionality of the feature space and hence the risk of overfitting, and allowing for an easier interpretation of the resulting machine learning model, due to the lower number of features [28].

The class distribution in training dataset T^TRAIN^ is highly unbalanced since we have 279 non-injury examples and just 7 injury examples. To adjust this imbalance we oversample the minority class in T^TRAIN^ by using the adaptive synthetic sampling approach (ADASYN; We use the ADASYN function provided by the publicly available Python package imblearn– http://scikit-learn.org/imbalanced-learn) [29]. The ADASYN algorithm generates examples of the minority class to equalize the distribution of classes, hence reducing the learning bias (See S7 Appendix). Finally, we use T^TRAIN^ to detect the best hyper parameters of a decision tree classifier DT (Step 2 in Fig 1).

#### Phase 2: Model construction and validation

We then split T^TEST^ into two folds, f_1_ and f_2_, in order to perform a stratified cross validation (step 3 in Fig 1; we use only two folds in order to not excessively reduce the minority class size). In this step, we oversample fold f_1_ by using ADASYN and test DT on the other fold f_2_ (which is not oversampled). For cross validation purposes, we perform again step 3 inverting f_1_ and f_2_. The goodness of the forecasting model is evaluated by four metrics (i.e., precision, recall, F1-score and AUC) described in S8 Appendix. Note that, for injury forecasting purposes, we are interested in achieving high values of precision and recall on class 1 (injury). Let us assume that a coach makes a decision about whether or not to “stop” a player based on the suggestion of the injury forecaster, i.e., the player skips next training session or game every time the forecaster’s prediction associated with the player’s current training session is 1 (injury). In this scenario, the forecaster’s precision indicates how much we can trust the predictions: the higher the precision, the more a classifier’s predictions are reliable, i.e., the probability that the player will actually get injured is high. Trusting an injury forecaster with low precision is risky as it means producing many false positives (i.e., false alarms) and frequently stopping players unnecessarily, a condition clubs want to avoid especially for the key players. The recall indicates the fraction of injuries the forecaster detects over the total number of injuries: the higher the recall the more injuries the forecaster can detect. An injury forecaster with low recall detects just a small fraction of the injuries, meaning that many players will attend next training session or game and actually get injured. Trusting a forecaster with a low recall is risky as it would misclassify many actual injuries as non-injuries.

We repeated the entire injury prediction approach (i.e., all the three steps in Fig 1) 10,000 times in order to assess its stability with respect to the choice of the injury examples in the two folds. For the sake of comparison, we implemented other injury forecasters based on the ACWR and the monotony (or MSWR) techniques, which are among the two most used techniques for injury risk estimation and prediction in professional soccer (see S2 Appendix and S3 Appendix for details). Moreover, we compare our injury forecaster with four baselines. Baseline *B*_1_ randomly assigns a class to an example by respecting the distribution of classes. Baseline *B*_2_ always assigns the non-injury class, while baseline *B*_3_ always assigns the injury class. Baseline *B*_4_ is a classifier which assigns class 1 (injury) if PI^(EWMA)^ > 0, and 0 (no injury) otherwise. We also compare DT with a Random Forest classifier (RF) and a Logit classifier (LR).

## Results

**Fig 2:**
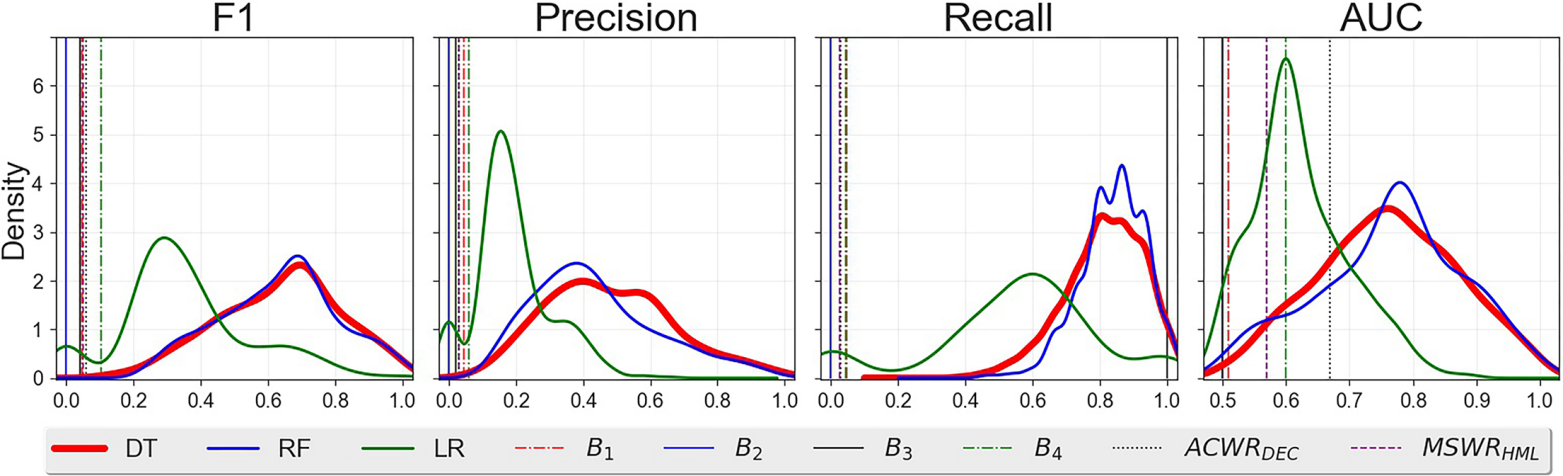
Classifiers performances. Distributions of the classifiers—DT, LR and RF—performances obtained testing the algorithms 10,000 times. This figure shows the performance of the baselines and the ACWR- and MSWR-based injury forecasters as well.

Table 2 compares the performance of DT with the performance of RF, LR, the ACWR and MSWR forecasters, and the four baselines. The results in Table 2 refer to the mean and the standard deviation of the evaluation metrics over 10,000 cross validation tasks. We find that DT has recall = 0.80±0.07 and precision = 0.50±0.11 on the injury class, meaning that the decision tree can predict almost all the injuries (80%) and that it correctly labels a training session as an injury in 50% of the cases. This is a significant improvement with respect to both the baselines *B*_1_, …,*B*_4,_ for which the maximum precision is about 6%, and the ACWR- and MSWR-based injury forecasters, for which the maximum precision is lower than 4%. RF has better recall but worse precision (recall = 0.87±0.05, precision = 0.41±0.08) that DT, while LR has much lower performance than the decision tree (Table 2). These results show that, typically, DT drastically reduces false alarms and hence scenarios where players are “stopped” unnecessarily before next game or training session. On the one hand, the distributions of the forecasters’ performances over the 10,000 tests indicate that the quality of the injury forecasting strongly depends on the type of injuries in the training set, which in turn depends on the different training and test split made in each trial (Fig 2). On the other hand, the higher performance detected by DT, compared to several baselines and the ACWR- and MSWR-based injury forecasters, shows that our approach outperforms state-of-the-art approaches and achieve good results in forecasting injuries. The results for DT without ADASYN and the oversampling process are presented in S9 Appendix.

**Table 2:** Performance of DT compared to RF, LR, the four baselines and the ACWR- and MSWR-based forecasters. For each forecaster we report precision, recall and F1 on the two classes and the overall AUC.

		precision	recall	F1	AUC
**DT**	***NI***	0.96±0.05	0.87±0.09	0.91±0.04	**0.76±0.12**
	***I***	**0.50±0.11**	**0.80±0.07**	**0.64±0.10**	
**RF**	***NI***	0.94±0.06	0.90±0.08	0.93±0.07	0.78±0.15
	***I***	0.41±0.08	0.87±0.05	0.65±0.08	
**LR**	***NI***	0.69±0.11	0.61±0.15	0.65±0.13	0.60±0.03
	***I***	0.18±0.03	0.60±0.08	0.31±0.06	
**B4**	***NI***	0.98	0.77	0.86	0.60
	***I***	0.04	0.43	0.07	
**B1**	***NI***	0.98	0.98	0.98	0.51
	***I***	0.06	0.05	0.05	
**B2**	***NI***	0.98	1.00	0.99	0.50
	***I***	0.00	0.00	0.00	
**B3**	***NI***	0.00	0.00	0.00	0.50
	***I***	0.02	1.00	0.04	
**C**^**(ACWR)**^_**DEC**_	***NI***	1.00	0.43	0.60	0.67
	***I***	0.04	0.91	0.07	
**C**^**(MSWR)**^_**HML**_	***NI***	0.98	0.80	0.88	0.57
	***I***	0.04	0.33	0.07	

As a further test of the forecasting potential of our approach we investigate the benefit of using our multi-dimensional injury forecaster in a real-world injury prevention scenario, where we assume that a club equips with appropriate GPS sensor technologies and starts recording training workload data since the first training session of the season (in other words, no data are available to the club before the beginning of the season). Assuming that we train the injury forecaster with new data every week, how many injuries the club can actually prevent throughout the season?

To answer this question we group the training sessions by week and proceed from the least recent to the most recent week. At training week *w*_*i*_ we first construct the dataset T_*i*_ consisting of all the training examples collected up to week *i*, oversampling the injury examples through ADASYN and reducing the feature space through RFECV. Then, we use T_*i*_ to train DT_*i*_, RF_*i*_, LR_*i*_, B_1,*i*, …,_B_4,*i*_, the ACWR- and MSWR-based forecasters and try to predict the injuries in week *w*_*i+1*_. At week *i*, we evaluate the accuracy of our approach by the cumulative F1-score, i.e., the F1-score computed by considering all the predictions made up to week *i* by the models DT_*6*_,…, DT_*i*_. Due to the initial scarcity of data, we start the forecasting task from week *w*_*6*_.

**Fig 3:**
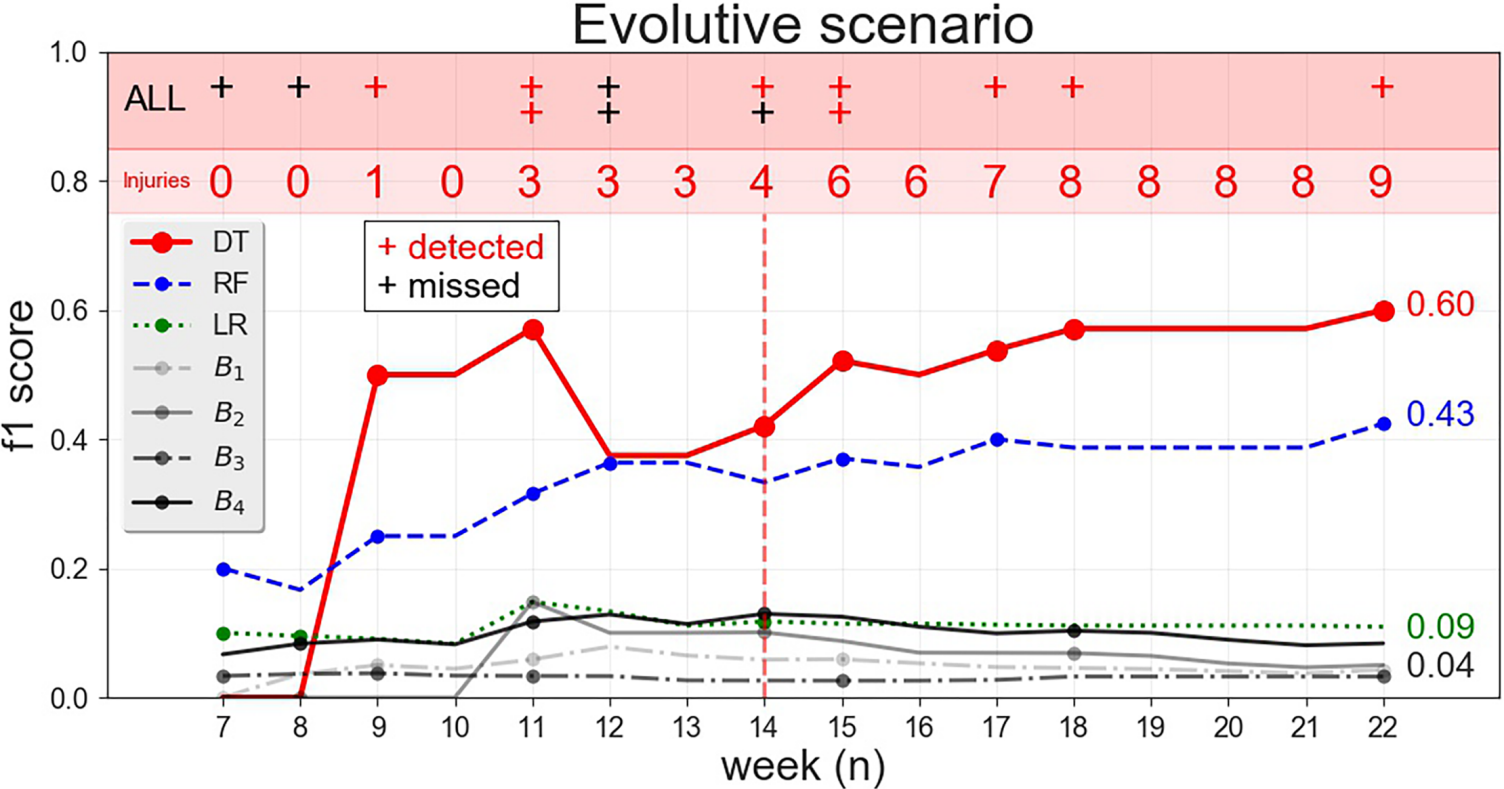
Performance of forecasters in the evolutive scenario. As the season goes by, we plot week by week the cumulative F1-score of the forecasters DT, RF, LR, B_1_, …, B_4_ trained on the data collected up to that week. Black crosses indicate injuries that not detect by DT, red crosses indicate injures correctly predicted by DT. For every week *i* we highlight in red the number of injuries detected by DT up to week *i*.

Fig 3 and S7 Table show the evolution of the cumulative F1-score and the feature extracted by RFECV as the season goes by, respectively. We find that in the first weeks DT has a poor predictive performance and misses many injuries (the black crosses in Fig 3). The predictive ability of DT improves significantly throughout the season: as more and more training and injury examples are collected, the forecasting model predicts most of the injuries in the second half of the season (the red crosses in Fig 3). We find that DT is the one performing the best, outperforming all the other models from week *w*_*14*_. In particular, DT detects 9 injuries out of 14 from *w*_*6*_ to the end of the season, resulting in F1-score = 0.60 and precision = 0.56. After an initial period of data collection, the injury forecaster becomes a useful tool to prevent the injuries of players and, by extracting the rules from the decision tree as we show in the next section, to understand the reasons behind the forecasted injuries as well as the injuries that are not detected by the model.

### Interpretation of the injury forecaster

A set of simple rules can be extracted from DT build on *w*_*21*_, allowing for the investigation of the reasons behind the observed injuries. These rules can be seen as a short handbook for coaches and athletic trainers, which can consult it to modify the training schedule and improve the players’ fitness.

**Fig 4:**
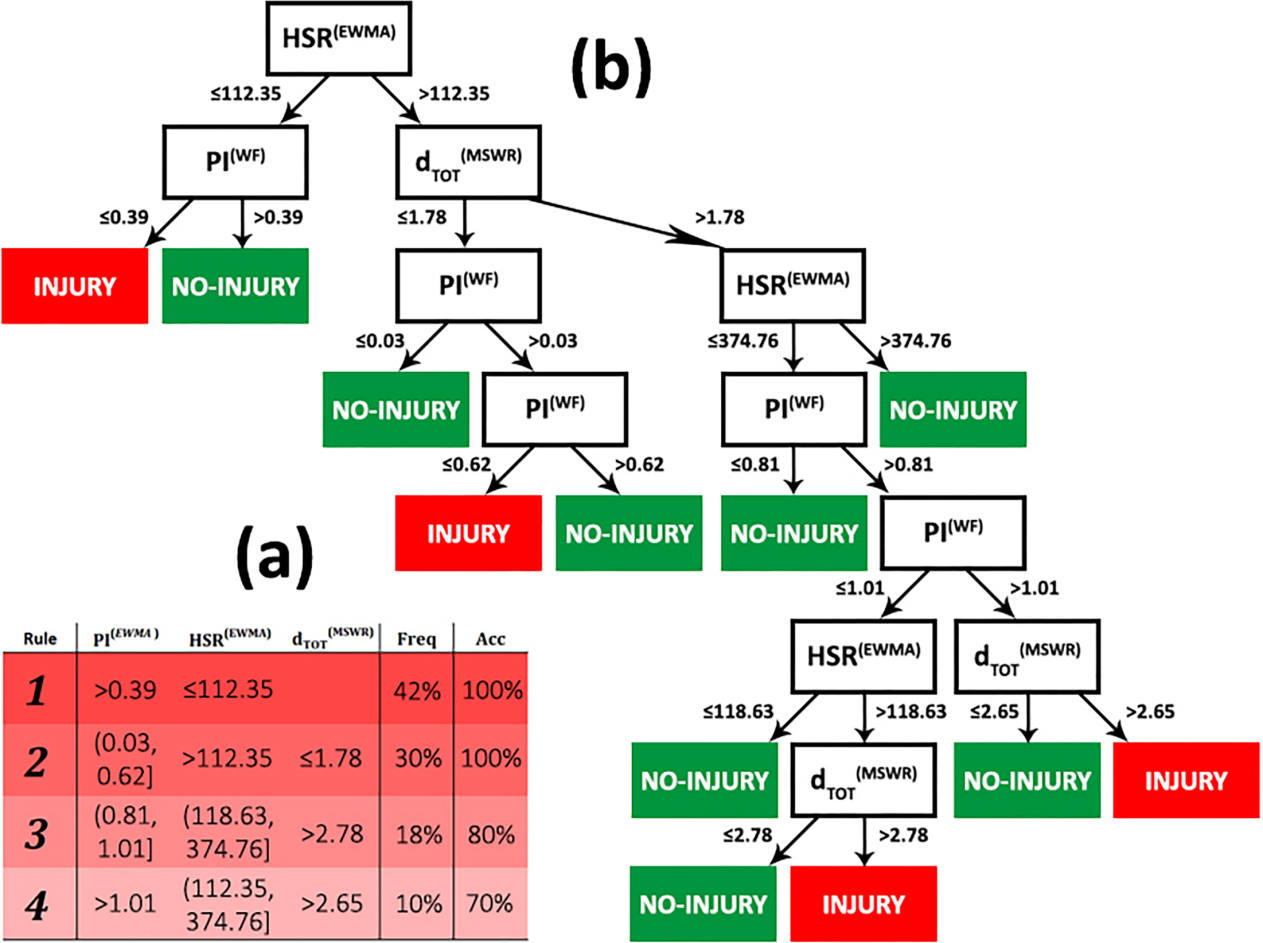
Interpretation of the multi-dimensional injury forecaster. **(a)** The six injury rules extracted from DT. For each rule we show the range of values of every feature, its frequency (Freq) and accuracy (Acc). **(b)** A schematic visualization of decision tree. Black boxes are decision nodes, green boxes are leaf nodes for class No-Injury, red boxes are leaf nodes for class Injury.

Fig 4B visualizes DT highlighting two types of node: decision nodes (black boxes) and leaf nodes (green or red boxes). Each decision node has two branches each indicating the next node to select depending on the range of values of the feature associated with the decision node. A leaf node represents the final prediction based on a player’s individual training session. There are two possible final decisions: Injury (red boxes) indicates that the player will get injured in next game or training session; or No-Injury (green boxes) otherwise. Given a feature vector describing a player’s training session, the prediction associated with it is obtained by following the path from the root of the tree down to a leaf node, through the decision nodes. Fig 4 shows the rules and the tree extracted from the DT built until *w*_*21*_. At the end of the season, the RFECV process selects just 3 features out of 55: PI^(EWMA)^, d_HSR_^(EWMA)^ and d_TOT_^(MSWR)^. The importances of these features in DT, computed as the mean decrease in Gini coefficient, are 0.71, 0.23 and 0.06, respectively [30].

As a practical example of application of these rules, let us consider a player’s training session with PI^(EWMA)^ = 0.28, *d*_*HSR*_^(EWMA)^ *=* 126.58 and *d*_*TOT*_^(MSWR)^ = 1.66, associated with an injury. This example is associated with rule 2 (Fig 4A), corresponding to the following decision path:

$$d_{HSR}{}^{\left(\mathrm{EWMA}\right)}>112.35\to d_{TOT}{}^{\left(\mathrm{MSWR}\right)}\leq 1.78\to PI^{\left(\mathrm{EWMA}\right)}>0.03\to PI^{\left(\mathrm{EWMA}\right)}\leq 0.68\to INJURY$$

From the rules in Fig 4A we summarize three main injury scenarios in DT:

1. a previous injury can lead to a new injury when a player has a HSR^(EWMA)^ (high speed running distance) lower than 112.35 (rule 1 in Fig 4A). This rule describes 42% of the injuries in the dataset and it is correct in 100% of the cases.
2. a previous injury can lead to a new injury when a player has a HSR^(EWMA)^ higher than 112.35 and a D_tot_^(MSWR)^ (total distance Monotony) three times lower than 1.78 (rule 2 in Fig 4A). This rule describes 30% of the injuries and has an accuracy of 100%.
3. a previous injury can lead a new injury when a player has a HSR^(EWMA)^ higher than 112.35 and a D_tot_^(MSWR)^ two and half times higher than the player’s average (rules 3 and 4 in Fig 4A). These rules have a cumulative frequency of 28% and a mean accuracy of 75±5%.

These scenarios suggest that coaches and athletic trainers must take care of the total distance and the distance at high speed running performed by the players who recently returned to play after an injury.

## Discussion

Our experiments produce three remarkable results. First, DT can detect around 80% of the injuries with about 50% precision, far better than the baselines and state-of-the-art injury risk estimation techniques (see Table 2). The decision tree’s false positive rate is small, indicating that it reduces the “false alarms”, i.e., situations where the classifier is wrong in predicting that an injury will happen. In professional soccer, false alarms are deprecable because the scarcity of players can negatively affect the performance of a team [2]. Our model also produces a moderate false negative rate, meaning that situations where a player that will get injured is classified as out of risk are infrequent.

Second remarkable results is that, in a real-world scenario of injury prevention where a club starts collecting the data for the first time and re-train the injury forecaster as the season goes by, the injury forecaster results in a cumulative F1-score = 0.60 on the injury class (Fig 3), much better than the baselines, RF and LR (Table 2). Throughout the season, the usage of the forecasting model allows for the prevention of more than half of the injuries. The forecasting ability of DT is affected by the initial period where data are scarce. This suggests that an initial period of data collection is needed in order to gather the adequate amount of data, and only then a reliable forecasting model can be trained on the collected data. The length of the data collection period depends on the club’s needs and strategy, including the frequency of training sessions and games, the frequency of injuries, the number of available players and the tolerated level of false alarms. Regarding this aspect, in our dataset, we observe that the performance of the classifiers stabilizes after 14 weeks of data collection (see Fig 3).

Third, in the evolutive scenario the features selected change as the season goes by (see S7 Table). This is probably due to the initial scarcity of data and to the type of injuries that have occurred up that a given moment. We observed that the just 3 out of 55 features are selected by the feature selection (PI^(EWMA)^, d_HSR_^(EWMA)^ and d_TOT_^(MSWR)^) after 14 weeks of data collection, and that these set of features remains stable for all subsequent weeks. Feature PI^(EWMA)^, the most important among the three and the only feature that is always selected as the season goes by (see S7 Table), reflects the temporal distance between a player’s current training session and his coming back to regular training after a previous injury. Less than half of the injuries detected by DT in the evolutive scenario happened immediately after the coming back to regular training of injured player. Furthermore, 60% of the injuries detected by DT happened long after a previous injury and are characterized by specific values of d_HSR_^(EWMA)^ and d_TOT_^(MSWR)^, which indicate that the a player’s kinematic variability affects his injury risk. It is worth to notice that the single feature PI^(EWMA)^ alone does not provide a significant predictive power, as the baseline B_4_, which is based on it, has a much lower accuracy than DT. It is hence the combination of the three features which allows us to predict when a player will get injured. Our results suggest that the club should take particular care of the first training sessions of players who come back to regular training after a previous injury, as in this conditions they are more likely to get injured again. In these first days and in the days long after the players return to regular physical activity, the club should control kinematic workloads, which can lead to injuries at specific values as well.

Injuries involve a great economic cost to the club, due to the expensive process of recovery and rehabilitation for the players. Injury prevention can reduce these costs by avoiding the injuries of players, which means improving the team’s performance and the player’s mental state as well as reducing the seasonal costs of medical care. We estimate that 139 days of absence during the seasons are due to injuries, corresponding to 6% of the working days. We observe that a player returned to regular physical activity within 5 days (i.e., 15 times out of 23 injuries), while only 6 times a player needed more than 5 days to recover. We use a method proposed in the literature [4] to estimate that the minimum total cost related to injuries that in this soccer club is 11,583 euros (139x83 euros = days of absence x minimal legal salary per day) corresponding to 3.81% of the salary cost of the club. If our model was used as the season goes by to stop the players for which an injury is predicted, the club could had been able to prevent 9 injuries out of 14 and save 8,881 euros (107x83 euros = day of absence x minimal legal salary per day), that represents a 77% decrease of injury costs.

## Conclusion

In this paper we proposed a multi-dimensional approach to injury forecasting in soccer, fully based on automatically collected GPS data and machine learning. As we showed, our injury forecaster provides a good trade-off between accuracy and interpretability, reducing the number of false alarms with respect to state-of-the-art approaches and at the same time providing a simple handbook of rules to understand the reasons behind the observed injuries. We showed that the forecaster can be profitably used early in the season, and that it allows the club to save a considerable part of the seasonal injury-related costs. Our approach opens a novel perspective on injury prevention, providing a methodology for evaluating and interpreting the complex relations between injury risk and training performance in professional soccer.

Our work can be extended in many directions. First, we can include performance features extracted from official games, where the player is exposed to the highest physical and psychological stress. Second, we can investigate the “transferability” of our approach from a club to another, i.e., if a forecaster trained on a set of players can be successfully applied to a distinct set of players, not used during the training process. In this case, it would be possible to exploit collective information to train a more powerful forecaster which includes training examples from different players, clubs, and leagues. Third, if data covering several seasons of a player’s activity are available, a distinct forecaster can be trained for each player by combining GPS data with other types of health data, such as heart rate, ventilation, and lactate.

## Supporting information

S1 Appendix Descriptive statistics of the workload features. (DOCX)

S2 Appendix The ACWR method. (DOCX)

S3 Appendix The MSWR method. (DOCX)

S4 Appendix Example of the training dataset construction. (DOCX)

S5 Appendix Exponential Weighted Moving Average (EWMA). (DOCX)

S6 Appendix Computation of PI^(WF)^. (DOCX)

S7 Appendix Adaptive synthetic sampling approach. (DOCX)

S8 Appendix Classifiers metrics assessment. (DOCX)

S9 Appendix Predictions results. (DOCX)

S1 Table Descriptive statistics of the 12 training workload features. We provide three categories of training workload features: kinematic features (blue), metabolic features (red) and mechanical features (green).
(DOCX)

S2 Table Performance of ACWR predictor. We report precision (prec), recall (rec), F1-score (F1) and Area Under the Curve (AUC) for the injury class and the non- injury class for all the predictors based on ACWR and MSWR. We also provide predictive performance of four baseline predictors *B*_1_, *B*_2_, *B*_3_ and *B*_4_.
(DOCX)

S3 Table Injury prediction report of ACWRq. We report precision (prec), recall (rec), F1-score (F1) and Area Under the Curve (AUC) for the injury class and the non-injury class for all the predictors defined on ACWR and monotony methodologies. We also provide predictive performance of four baseline predictors *B*_1_, *B*_2_, *B*_3_ and *B*_4_.
(DOCX)

S4 Table Performance of MSWR predictor. We report precision (prec), recall (rec), F1-score (F1) and Area Under the Curve (AUC) for the injury class and the non- injury class for all the predictors based on ACWR and MSWR. We also provide predictive performance of four baseline predictors *B*_1_, *B*_2_, *B*_3_ and *B*_4_.
(DOCX)

S5 Table PI^(WF)^ values after n training days (i.e., *n* = 1, …, 6) since the return of a player to regular training. We report the values for different n of previous injuries (i.e., *n* = 1, …, 4). PI_*i*_ is the number of training days long after players return to regular physical activity. 6+ indicates values for 6 and more than 6 days.
(DOCX)

S6 Table Performance of the classifiers on T^(ADA)^, T and T^(REF)^. For each classifier, we report the precision (prec), recall (rec) and F1-score (F1) on the two classes and the overall AUC.
(DOCX)

S7 Table Feature selection real-world scenario. Features extracted by RFECV in each T_i_ built as the season went by.
(DOCX)

S1 Fig Distribution of workload features. We provide three categories of training workload features: kinematic features (blue), metabolic features (red) and mechanical features (green).
(TIF)

S2 Fig Injury risk in ACWR groups. The plots show Injury Likelihood (IL) for pre- defined ACWR groups [29], for every of the 12 training workload features considered in our study. Bars are colored according to feature categorization defined in Table 1.
(TIF)

S3 Fig Injury likelihood in ACWR groups. The plots show IL for the ACWR groups defined the quantiles of the distribution, for every of the 12 training workload features considered in our study. We provide three categories of training workload features: kinematic features (blue), metabolic features (red) and mechanical features (green).
(TIF)

S4 Fig Injury risk in MSWR groups. The plots show the Injury Likelihood (IL) for the MSWR groups for every of the 12 training workload features considered in our study. Bars are colored according to feature categorization defined in Table 1.
(TIF)

S5 Fig We plot the AUC and F1-score of EWMA with span = 1, …, 10 in CALL. The red line reflects the best span to injury prediction.
(TIF)

## References

[1] Hägglund M, Waldén M, Magnusson H, Kristenson H, Bengtsson H, Exstrand J. Injuries affect team performance negatively in professional football: an 11-year follow-up of the UEFA Champions League injury study. British Journal of Sports Medicine, 10.1136/bjsports-2013-092215, 2013 23645832

[2] Hurley OA. Impact of Player Injuries on Teams’ Mental States, and Subsequent Performances, at the Rugby World Cup 2015. Frontiers in Psychology 7:807, 10.3389/fpsyg.2016.00807 27375511 PMC4891352

[3] Lehmann EE, Schulze GG. What Does it Take to be a Star?–The Role of Performance and the Media for German Soccer Players. Applied Economics Quarterly 54:1, pp. 59–70, 10.3790/aeq.54.1.59, 2008.

[4] Fernández-Cuevas I., Gomez-Carmona P, Sillero-Quintana M, Noya-Salces J, Arnaiz-Lastras J, Pastor-Barrón A. Economic costs estimation of soccer injuries in first and second Spanish division professional teams. 15th Annual Congress of the European College of Sport Sciences ECSS, 23th 26th june. 2010.

[5] Gudmundsson H, Horton M. Spatio-Temporal Analysis of Team Sports, ACM Computing Surveys 50, 2, Article 22 (4 2017), 34 pages. 10.1145/3054132.

[6] Stein M, Janetzko H, Seebacher D, Jäger A, Nagel M, Hölsch J, et al How to Make Sense of Team Sport Data: From Acquisition to Data Modeling and Research Aspects. Data, 2:1, 2, 10.3390/data2010002, 2017.

[7] Rossi A, Savino M, Perri E, Aliberti G, Trecroci A, Iaia M. Characterization of in-season elite football trainings by GPS features: The Identity Card of a Short-Term Football Training Cycle. 16th IEEE International Conference on Data Mining Workshops, pp. 160–166, 10.1109/ICDMW.2016.0030, 2016

[8] Pappalardo L, Cintia P. Quantifying the relation between performance and success in soccer, Advances in Complex Systems, 20 (4), 10.1142/S021952591750014X, 2017.

[9] Cintia P, Pappalardo L, Pedreschi D, Giannotti F, Malvaldi M. The harsh rule of the goals: data-driven performance indicators for football teams, In Proceedings of the 2015 IEEE International Conference on Data Science and Advanced Analytics (DSAA’2015), 10.1109/DSAA.2015.7344823, 2015.

[10] Brink MS, Visscher C, Arends S, Zwerver J, Post WJ, Lemmink KA. Monitoring stress and recovery: new insights for the prevention of injuries and illnesses in elite youth soccer players. Br J Sports Med. 2010;44: 809–15. 10.1136/bjsm.2009.069476 20511621

[11] Ehrmann FE, Duncan CS, Sindhusake D, Franzsen WN, Greene DA. GPS and injury prevention in professional soccer. J Strength Cond Res. 2015;30:306–307. 10.1519/JSC.0000000000001093 26200191

[12] Venturelli M, Schena F, Zanolla L, Bishop D. Injury risk factors in young soccer players detected by a multivariate survival model. Journal of Science and Medicine in Sport. 2011;14:293–298. 10.1016/j.jsams.2011.02.013 21474378

[13] Kirkendall DT, Dvorak J. Effective Injury Prevention in Soccer. The physician and sports medicine, 38:1, 10.3810/psm.2010.04.1772, 2010. 20424412

[14] Gabbett TJ. The development and application of an injury prediction model for noncontact, soft-tissue injuries in elite collision sport athletes. The Journal of Strength & Conditioning Research. 2010;24(10):2593–2603. 10.1519/JSC.0b013e3181f19da4 20847703

[15] Gabbett TJ, Ullah S. Relationship between running loads and soft-tissue injury in elite team sport athletes. J Strength Cond Res. 2012;26: 953–960. 10.1519/JSC.0b013e3182302023 22323001

[16] Rogalski B, Dawson B, Heasman J, Gabbett TJ. Training and game loads and injury risk in elite Australian footballers. J Sci Med Sport. 2013;16: 499–503. 10.1016/j.jsams.2012.12.004 23333045

[17] Gabbett TJ. The training-injury prevention paradox: should athletes be training smarter and harder? Br J Sports Med. 2016 10.1136/bjsports-2015-095788 26758673 PMC4789704

[18] Anderson L, Triplett-McBride T, Foster C, Doberstein S, Brice G. Impact of training patterns on incidence of illness and injury during a women’s collegiate basketball season. The Journal of Strength & Conditioning Research. 2003; 17: 734–738. 10.1519/00124278-200311000-00018 14636112

[19] Gabbett TJ. Reductions in pre-season training loads reduce training injury rates in rugby league players. British Journal of Sports Medicine. 2004;38: 74–749. 10.1136/bjsm.2003.005181 15562171 PMC1725000

[20] Hulin BT, Gabbett TJ, Blanch P, Chapman P, Bailey D, Orchard JV. Spikes in acute workload are associated with increased injury risk in elite cricket fast bowlers. Br J Sports Med. 2014;48:708–712. 10.1136/bjsports-2013-092524 23962877

[21] Foster C. Monitoring training in athletes with reference to overtraining syndrome. Med Sci Sports Exerc. 1998;30:1164–1168. 10.1097/00005768-199807000-00023 9662690

[22] Talukder H, Vincent T, Foster G, Hu C, Huerta J, Kumar A, et al. Preventing in-game injuries for NBA players. MIT Sloan Analytics Conference. Boston; 2016.

[23] Kampakis S. Predictive modeling of football injuries, Phd Thesis, University College London, 2016

[24] Hagglund M, Walden M, Bahr R, Ekstrand J. Methods for epidemiological study of injuries to professional football players: developing the UEFA model. British Journal of Sports Medicine, 39:6, 340–346, 10.1136/bjsm.2005.018267, 2005 15911603 PMC1725241

[25] Duncan MJ, Badland HM, Mummery WK. Applying GPS to enhance understanding of transport-related physical activity. Journal of Science and Medicine in Sport. 2009;12: 549–556. 10.1016/j.jsams.2008.10.010 19237315

[26] Murray NB, Gabbett TJ, Townshend AD, Blanch P. Calculation acute:chronic workload ratios using exponential weighted moving averages provides a more sensitive indicator of injury likelihood than rolling averages. Br J Sports Med. 2016 10.1136/bjsports-2016-097152 28003238

[27] Guyon I, Weston J, Barnhill S, Vapnik V. Gene Selection for Cancer Classification Using Support Vector Machines. Machine Learning 46, 2002 10.1023/A:1012487302797

[28] James G, Witten D, Hastie T, Tibshirani R. An Introduction to Statistical Learning New York, NY: Springer New York; 2013.

[29] He H, Bai Y, Garcia EA, Li S. ADASYN: Adaptive synthetic sampling approach for imbalanced learning. 2008 IEEE International Joint Conference on Neural Networks.

[30] Kazemitabar J, Amini A, Bloniarz A, Talwalkar A. Variable Importance using Decision Trees. 31st Conference on Neural Information Processing Systems (NIPS 2017), Long Beach, CA, USA.

