# Supplementary material for: Effective injury forecasting in soccer with GPS training data and machine learning
Source: PLoS One. 2018 Jul 25;13(7):e0201264. doi: 10.1371/journal.pone.0201264 (PMC6059460; doi:10.1371/journal.pone.0201264)
Supplement: S1 Appendix — (DOCX) [file pone.0201264.s001.docx]

**S1 Appendix. Descriptive statistics of the workload features**

S1 Table shows the average (AVG) and the standard deviation (SD) of the distributions of the 12 training workload features considered in our study. We assess the normality of the distributions by using the Shapiro-Wilks’ Normality test (SW) and observe that none of them is normally distributed (see S1 Table). Indeed, by a visual inspection of the distributions, we observe that they tend to be bimodal and right skewed (S1 Fig).
