# Supplementary material for: Effective injury forecasting in soccer with GPS training data and machine learning
Source: PLoS One. 2018 Jul 25;13(7):e0201264. doi: 10.1371/journal.pone.0201264 (PMC6059460; doi:10.1371/journal.pone.0201264)
Supplement: S2 Appendix — (DOCX) [file pone.0201264.s002.docx]

**S2 Appendix. The ACWR method**

The Acute Chronic Workload ratio (ACWR), defined as the ratio between a player’s acute workload and his chronic workload [14, 20, 26], is one of the most used technique for injury risk estimation in professional soccer [31]. A player’s acute and chronic workloads are estimated by the exponential weighted moving average of a single workload feature *h* in the previous 7 days and 28 days, respectively. The player’s ACWR is then used to estimate his injury likelihood [26].

We reproduce the ACWR methodology for each of the 12 workload features used in our study, using the ACWR groups suggested by Murray et al. [26]. They compute the ACWR for a set of workload features and categorize the players’ training sessions with five groups: (1) ACWR < 0.49 (very low); (2) ACWR [0.50, 0.99] (low); (3) ACWR [1.00, 1.49] (moderate); (4) ACWR [1.50, 1.99] (high); (5) ACWR > 2.00 (very high). Then, the injury likelihood (IL) is estimated in every ACWR group as the ratio between the number of players who get injured after the training session assigned to that ACWR group and the number of players who do not. Murray et al. [26] observe that players whose training sessions result in ACWR > 2 have a higher injury risk than the players in the other groups (i.e., a high IL). In contrast with the literature, we do not find any individual training session resulting in ACWR > 2 (see S2 Fig), while we observe that players whose individual training sessions result in ACWR < 1 have the highest injury risk (S2 Fig).

Additionally, we explore the usability in practice of the ACWR method by constructing injury forecasting models based on the ACWR method. In particular, given a player’s training session, a predictive model C*_h_*^(ACWR)^ predicts whether or not the player will get injured during next game or training session based on the value of workload feature *h*. If considering feature *h* the individual training session results in ACWR < 1, C*_h_*^(ACWR)^ forecasts an injury (class 1) otherwise it forecasts a non-injury (class 0). We find that C*_h_*^(ACWR)^ has in average a high recall (0.80 ± 0.08) but a very low precision (0.03 ± 0.003), denoting the presence of a high rate of false alarms, as in average the models wrongly predict an injury in 97% of the cases. Moreover, we combine the ACWR forecasters in three ways:

- the predictive model C_(vote)_ predicts a player will get injured if his training session results in ACWR < 1 for the majority of the workload features;
- the predictive model C_(all)_ predicts that a player will get injured if his training session results in ACWR < 1 for all the workload features;
- the predictive model C_(one)_ predicts an injury if ACWR < 1 for at least one workload feature.

S2 Table reports the accuracy of C_(vote)_, C_(all)_, C_(one)_. Only C_(vote)_ achieves a slightly better performance, in terms of precision on the injury class, than the ACWR forecasters based on the single features. We compare the predictors with four baselines. Baseline B_1_ randomly assigns a class to an example by respecting the distribution of classes. Baseline B_2_ always assigns the majority class (i.e., class 0, a non-injury), while baseline B_3_ always assigns the minority class (i.e., class 1, injury). Baseline B_4_ is a classifier which assigns class 1 (injury) if the exponentially weighted average of variable PI > 0 (see S5 Appendix), and 0 (no injury) otherwise. Although the 15 ACWR forecasters are significantly better than the baseline classifiers in terms of recall on the injury class, our results suggest that a predictor based on ACWR is not usable in practice due to its low precision. In a scenario where a coach or an athletic trainer bases his decisions on the suggestions of the predictors, in the vast majority of the cases he would generate “false alarms” by stopping a player with no risk of injury, which is not a practical solution to injury prevention in professional soccer.

We also replicate the experiment by using ACWR groups defined by the quintiles of the ACWR distribution instead of the pre-defined groups proposed by Murray et al. [26]. S3 Fig shows the injury likelihood (IL) for every ACWR quintile for all the 12 workload features. We observe that the groups with low ACWR are associated to the highest injury risk, substantially confirming the experiments made using predefined ACWR groups. We construct predictors C*_h_*^(ACWRq)^ following the same strategy as above but using quantiles instead of predefined groups. S3 Table visualizes the results of classification, which are similar to those presented in the manuscript for the pre-defined ACWR groups: the predictors are a little usable in practice due to their too low precision.

**Reference**

[31] Fuller CW, Ekstrand J, Junge A, Andersen TE, Bahr R, Dvorak J, et al. Consensus statement on injury definitions and data collection procedures in studies of football (soccer) injuries. British Journal of Sports Medicine, 40, pp. 193-201, doi:http://dx.doi.org/10.1136/bjsm.2005.025270, 2006.
