# Supplementary material for: Effective injury forecasting in soccer with GPS training data and machine learning
Source: PLoS One. 2018 Jul 25;13(7):e0201264. doi: 10.1371/journal.pone.0201264 (PMC6059460; doi:10.1371/journal.pone.0201264)
Supplement: S3 Appendix — (DOCX) [file pone.0201264.s003.docx]

**S3 Appendix. The MSWR method**

Another widely used method for injury risk estimation is the Mean Standard deviation Workload Ratio (MSWR, or Monotony), defined as the ratio between the mean and the standard deviation of a single player’s workload feature *h* obtained in one week [10, 18, 21]. High MSWR values are generally associated with negative game performance and high injury risk [21].

We investigate the relation between MSWR and injury risk by grouping the individual training sessions into quintiles according to the distribution of the workload features. For every quintile, we compute the corresponding injury likelihood (IL). We observe that high MSWR values are related to high injury risk for the majority of workload features, substantially confirming results observed in the literature by Foster et al. [30] (see S4 Fig).

As done for ACWR, we explore the usability in practice of MSWR by constructing 12 predictive models based on the 12 training workload features. Given a player’s training session, every predictive model C_MSWR_ predicts whether or not the player will get injured during next game or training session based on the value of workload feature *h*. If considering feature *h* the individual training session is associated with the MSWR group with the highest injury risk, model C_MSWR_ predicts an injury (class 1), otherwise it predicts a non-injury (class 0). We find that C*_h_*^(MSWR)^ has in average both a low recall (0.10 ± 0.10) and a low precision (mean is 0.03 ± 0.03, see S4 Table). Moreover, we construct three combined models – C_(vote)_, C_(all)_ and C_(one)_ – and we observe that they have poor accuracy in detecting the injury class (S4 Table). In particular, the MSWR predictors have predictive power comparable to the ACWR predictors.
