# Supplementary material for: Effective injury forecasting in soccer with GPS training data and machine learning
Source: PLoS One. 2018 Jul 25;13(7):e0201264. doi: 10.1371/journal.pone.0201264 (PMC6059460; doi:10.1371/journal.pone.0201264)
Supplement: S4 Appendix — (DOCX) [file pone.0201264.s004.docx]

**S4 Appendix. Example of the training dataset construction**

Let us consider a toy dataset consisting of a portion of the training sessions of a player *D* = {*s*_6_, *s*_7_, *s*_8_, *s*_9_} where the last session (*s*_9_) is associated with an injury, i.e., the player will get injured during training session *s*_10_. We construct the training dataset *T* as follows:

1. We create a new example in dataset *T* for each training session in *D*, by computing 42 player’s workload features. Every example is described by a vector of length 42, *m_i_* = (*h*_1_, … , *h*_42_). All the four vectors compose matrix *F* = (*m*_1_*, m*_2_*, m*_3_*, m*_4_);
2. Since the first three training sessions are not associated with injuries, the first three examples *m*_1_*, m*_2_*, m*_3_ have injury label 0. The last example *m*_4_ has injury label 1 since it is associated with an injury. Therefore, the labels vector is hence *c* = (0, 0, 0, 1), indicating that the first three examples are not associated with an injury while the last training session produces an injury. The training dataset based on *D* and feature set *all* is finally *T*= (*F*, *c*).
