# Supplementary material for: Effective injury forecasting in soccer with GPS training data and machine learning
Source: PLoS One. 2018 Jul 25;13(7):e0201264. doi: 10.1371/journal.pone.0201264 (PMC6059460; doi:10.1371/journal.pone.0201264)
Supplement: S5 Appendix — (DOCX) [file pone.0201264.s005.docx]

**S5 Appendix. Exponential Weighted Moving Average (EWMA)**

To consider the recent training workload of a player, we compute the exponential weighted moving average (ewma) of his most recent training sessions. The ewma decreases exponentially the weights of the values according to their recency [32, 33], i.e., the more recent a value is the more it is weighted in an exponential function according to a decay α = 2/(span 1). In accordance with the exponential function, the moving average is computed as:

EWMA*_t_* = α[x_t_−(x_t−1_+(1−α)^2^x_t−2_+…+(1−α)^n−1^x_t−n_)]+x_t_

We vary span = 1, … , 10 to detect the value leading to the best classification performance. We hence train a decision tree on the feature set all by using every of the ten span values. Fig 10 shows the cross-validated AUC and F1-score of the decision tree DT^(RFE)^ varying the value of span. We observe that a 6 training span is the best predictive window to injury prediction in our dataset (S5 Fig).

**Reference**

[32] Lowry CA, Woodall WH, Champ CW, Rigdon SE. A Multivariate Exponentially Weighted Moving Average Control Chart. Technometrics. Technometrics. 1992;34: 4653. doi: 10.2307/1269551.

[33] Lucas JM, Saccucci MS. Exponentially Weighted Moving Average Control Schemes: Properties and Enhancements. Technometrics. 1990;32: 112. doi: 10.2307/1269835.
