# Supplementary material for: Effective injury forecasting in soccer with GPS training data and machine learning
Source: PLoS One. 2018 Jul 25;13(7):e0201264. doi: 10.1371/journal.pone.0201264 (PMC6059460; doi:10.1371/journal.pone.0201264)
Supplement: S6 Appendix — (DOCX) [file pone.0201264.s006.docx]

**S6 Appendix. Computation of PI^(WF)^**

To take into account the injury history of a player, we compute the EWMA of the number of injuries in previous weeks. PI^(WF)^ reflects the temporal distance between a player’s training session and the return of the player to regular training after an injury. PI^(WF)^ = 0 represents players who never got injured in the past. PI^(WF)^ > 0 represents players who get injured at least once in the past. S5 Table provides specific PI^(WF)^ thresholds in players incurred from 1 to 4 previous injuries. For example, PI^(WF)^ = 0.50 reflects a training performed by a player 3 days since his return to regular training after an injury.
