# Supplementary material for: Effective injury forecasting in soccer with GPS training data and machine learning
Source: PLoS One. 2018 Jul 25;13(7):e0201264. doi: 10.1371/journal.pone.0201264 (PMC6059460; doi:10.1371/journal.pone.0201264)
Supplement: S7 Appendix — (DOCX) [file pone.0201264.s007.docx]

**S7 Appendix. Adaptive synthetic sampling approach**

For each new instance of the minority class in T^TRAIN^, ADASYN automatically decides the number of synthetic samples that need to be generated according to a density distribution of the majority class in T^TRAIN^. The dataset resulting from ADASYN provides a balanced representation of the data distribution. Each new example is computed as x*_i_*+(x*_zi_*-x*_i_*)λ where (x*_zi_*-x*_i_*) is the difference vector in a n-dimensional space and λ is an arbitrary number between 0 and 1 randomly assigned in each new example [29].
