# Supplementary material for: Effective injury forecasting in soccer with GPS training data and machine learning
Source: PLoS One. 2018 Jul 25;13(7):e0201264. doi: 10.1371/journal.pone.0201264 (PMC6059460; doi:10.1371/journal.pone.0201264)
Supplement: S8 Appendix — (DOCX) [file pone.0201264.s008.docx]

**S8 Appendix. Classifiers metrics assessment**

1. **Precision**: prec = TP/(TP+FP), where TP and FP are true positives and false positives resulting from classification, where we consider the injury class (1) as the positive class. Given a class, precision indicates the fraction of examples that the classifier correctly classifies over the number of all examples the classifier assigns to that class;
2. **Recall**: rec = TP/(TP + FN), where FN are false negatives resulting from classification. Given a class, the recall indicates the ratio of examples of a given class correctly classified by the classifier;
3. **F1-measure**: F1 = 2(prec * rec)/(prec + rec). This measure is the harmonic mean of precision and recall, which in our case coincides with the square of the geometric mean divided by the arithmetic mean;
4. **Area Under the Curve (AUC)**: the probability that a classifier will rank a randomly chosen positive instance higher than a randomly chosen negative one (assuming “positive” ranks higher than “negative”). An AUC close to 1 represents an accurate classification, while an AUC close to 0.5 represents a classification close to randomness.
