# Supplementary material for: Effective injury forecasting in soccer with GPS training data and machine learning
Source: PLoS One. 2018 Jul 25;13(7):e0201264. doi: 10.1371/journal.pone.0201264 (PMC6059460; doi:10.1371/journal.pone.0201264)
Supplement: S9 Appendix — (DOCX) [file pone.0201264.s009.docx]

**S9 Appendix. Predictions results**

**Oversampling without feature selection**

Here we provide the performance of the classifiers trained without the feature selection process (i.e., we use the same approach provide in Figure 1 without the feature selection process shows in the step 2).

We find that DT^(ADA)^ has precision=0.88 and recall=0.92 on the injury class. Although DT^(ADA)^ (i.e., scenario without feature selection) has a performance comparable to DT^(ADA+RFE)^ (i.e., scenario with feature selection), the latter uses just 3 features out of 55, resulting in a decision tree much easier to interpret and understand.

**No-oversampling without feature selection**

Here we provide the performance of the classifiers trained on the unbalanced training dataset T (931 no-injury and 23 injury examples). On this dataset we train DT^(T)^, RF^(T)^ and LR^(T)^. We validate the classifiers with a 3-fold stratified cross-validation strategy: the real dataset is divided into 3 parts or folds and, for each fold, we use the 10% of the target values as test set, and the remaining 90% as training set.

DT^(T)^ has precision=0.70 and recall=0.47 on the injury class. RF^(T)^ provides just a tiny improvement in terms of recall, but not in precision (precision=0.88, recall=0.60), while LR^(T)^ has much lower performance (precision=0.58, recall 0.33) than DT^(T)^.

**No-oversampling with feature selection**

Here we provide the performance of the classifiers trained on the unbalanced training dataset T (931 no-injury and 23 injury examples) on which we perform feature selection by RFECV to determine the most relevant features for classification. We detected that PI^(EWMA)^, d_HML_^(MSWR)^ and Dec_2_^(EWMA)^ are the most important features. Second, on the new training dataset T^(RFE)^ derived from the feature selection, we train DT^(RFE)^, RF^(RFE)^ and LR^(RFE)^. We validate the classifiers with a 3-fold stratified cross-validation strategy: the real dataset is divided into 3 parts or folds and, for each fold, we use the 10% of the target values as test set, and the remaining 90% as training set.

DT^(RFE)^ is able to detect 56% of the injuries with a precision of 74%. RF^(RFE)^ provides just a tiny improvement in terms of recall, but not in precision (precision=0.78, recall 0.58), while LR^(ADA)^ has much lower performance (precision=0.73, recall 0.48) than DT^(RFE)^.
