# Supplementary material for: Effective injury forecasting in soccer with GPS training data and machine learning
Source: PLoS One. 2018 Jul 25;13(7):e0201264. doi: 10.1371/journal.pone.0201264 (PMC6059460; doi:10.1371/journal.pone.0201264)
Supplement: S1 Table — We provide three categories of training workload features: kinematic features (blue), metabolic features (red) and mechanical features (green). (DOCX) [file pone.0201264.s010.docx]

|  | **AVG** | **SD** | **SW** |  |
| --- | --- | --- | --- | --- |
| d_TOT_ | 3882.94 | 1633.21 | *<*0.01 |  |
| d_HSR_ | 133.22 | 66.41 | *<*0.01 |  |
| d_MET_ | 1151.99 | 694.25 | *<*0.01 |  |
| d_HML_ | 543.89 | 339.64 | *<*0.01 |  |
| d_HML/m_ | 8.70 | 6.09 | *<*0.01 |  |
| d_EXP_ | 410.67 | 221.29 | *<*0.01 |  |
| Acc_2_ | 64.26 | 31.72 | *<*0.01 |  |
| Acc_3_ | 16.16 | 10.97 | *<*0.01 |  |
| Dec_2_ | 62.44 | 33.09 | *<*0.01 |  |
| Dec_3_ | 19.14 | 12.78 | *<*0.01 |  |
| DSL | 117.98 | 78.52 | *<*0.01 |  |
| FI | 0.63 | 0.31 | *<*0.01 |  |
| SD = Standard Deviation;  SW = Shapiro-Wilks’ Normality test. | | | | |
