# Supplementary material for: Effective injury forecasting in soccer with GPS training data and machine learning
Source: PLoS One. 2018 Jul 25;13(7):e0201264. doi: 10.1371/journal.pone.0201264 (PMC6059460; doi:10.1371/journal.pone.0201264)
Supplement: S4 Table — We report precision (prec), recall (rec), F1-score (F1) and Area Under the Curve (AUC) for the injury class and the non- injury class for all the predictors based on ACWR and MSWR. We also provide predictive performance of four baseline predictors B1, B2, B3 and B4. (DOCX) [file pone.0201264.s013.docx]

| **MSWR** | **class** | **prec** | **rec** | **F1** | **AUC** |
| --- | --- | --- | --- | --- | --- |
| C*_d_*_TOT_ | 0 | 0.98 | 0.80 | 0.88 | 0.57 |
|  | 1 | 0.04 | 0.33 | 0.07 |  |
| C*_d_*_HSR_ | 0 | 0.98 | 1.00 | 0.99 | 0.50 |
|  | 1 | 0.00 | 0.00 | 0.00 |  |
| C*_d_*_MET_ | 0 | 0.98 | 0.95 | 0.96 | 0.55 |
|  | 1 | 0.06 | 0.14 | 0.09 |  |
| C*_d_*_HML_ | 0 | 0.98 | 0.96 | 0.97 | 0.53 |
|  | 1 | 0.06 | 0.10 | 0.07 |  |
| C*_d_*_HML_*_/m_* | 0 | 0.98 | 0.96 | 0.97 | 0.55 |
|  | 1 | 0.08 | 0.14 | 0.10 |  |
| C*_d_*_EXP_ | 0 | 0.98 | 0.94 | 0.96 | 0.49 |
|  | 1 | 0.02 | 0.05 | 0.03 |  |
| C*_Acc_*_2_ | 0 | 0.98 | 0.93 | 0.95 | 0.46 |
|  | 1 | 0.00 | 0.00 | 0.00 |  |
| C*_Acc_*_3_ | 0 | 0.98 | 0.98 | 0.98 | 0.49 |
|  | 1 | 0.00 | 0.00 | 0.00 |  |
| C*_Dec_*_2_ | 0 | 0.98 | 0.94 | 0.96 | 0.52 |
|  | 1 | 0.04 | 0.10 | 0.05 |  |
| C*_Dec_*_3_ | 0 | 0.98 | 0.99 | 0.98 | 0.49 |
|  | 1 | 0.00 | 0.00 | 0.00 |  |
| C_DSL_ | 0 | 0.98 | 0.97 | 0.97 | 0.48 |
|  | 1 | 0.00 | 0.00 | 0.00 |  |
| C_FI_ | 0 | 0.98 | 0.72 | 0.83 | 0.50 |
|  | 1 | 0.03 | 0.29 | 0.04 |  |
| C_one_ | 0 | 0.98 | 0.56 | 0.71 | 0.54 |
|  | 1 | 0.03 | 0.52 | 0.05 |  |
| C_vote_ | 0 | 0.97 | 0.99 | 0.98 | 0.49 |
|  | 1 | 0.00 | 0.00 | 0.00 |  |
| C_all_ | 0 | 0.97 | 1.00 | 0.99 | 0.50 |
|  | 1 | 0.00 | 0.00 | 0.00 |  |
| *B*_1_ | 0 | 0.98 | 0.98 | 0.98 | 0.51 |
|  | 1 | 0.06 | 0.05 | 0.05 |  |
| *B*_2_ | 0 | 0.98 | 1.00 | 0.99 | 0.50 |
|  | 1 | 0.00 | 0.00 | 0.00 |  |
| *B*_3_ | 0 | 0.00 | 0.00 | 0.00 | 0.50 |
|  | 1 | 0.02 | 1.00 | 0.04 |  |
| *B*_4_ | 0 | 0.98 | 0.77 | 0.86 | 0.60 |
|  | 1 | 0.04 | 0.43 | 0.07 |  |
