# Supplementary material for: Effective injury forecasting in soccer with GPS training data and machine learning
Source: PLoS One. 2018 Jul 25;13(7):e0201264. doi: 10.1371/journal.pone.0201264 (PMC6059460; doi:10.1371/journal.pone.0201264)
Supplement: S5 Table — We report the values for different n of previous injuries (i.e., n = 1, …, 4). PIi is the number of training days long after players return to regular physical activity. 6+ indicates values for 6 and more than 6 days. (DOCX) [file pone.0201264.s014.docx]

|  | **PI***_i_* | | | | | | |
| --- | --- | --- | --- | --- | --- | --- | --- |
| **injuries** | **1** | **2** | **3** | **4** | **5** | **6+** |  |
| **1** | 0.29 | 0.49 | 0.64 | 0.74 | 0.81 | *>* 0*.*86 |  |
| **2** | 1.27 | 1.48 | 1.63 | 1.74 | 1.81 | *>* 1*.*86 |  |
| **3** | 2.27 | 2.46 | 2.62 | 2.72 | 2.8 | *>* 2*.*85 |  |
| **4** | 3.25 | 3.46 | 3.53 | 3.66 | 3.76 | *>* 3*.*83 |  |
