# Supplementary material for: Effective injury forecasting in soccer with GPS training data and machine learning
Source: PLoS One. 2018 Jul 25;13(7):e0201264. doi: 10.1371/journal.pone.0201264 (PMC6059460; doi:10.1371/journal.pone.0201264)
Supplement: S6 Table — For each classifier, we report the precision (prec), recall (rec) and F1-score (F1) on the two classes and the overall AUC. (DOCX) [file pone.0201264.s015.docx]

|  |  | **T^(ADA)^** | | | | **T** | | | | **T^(RFE)^** | | | |
| --- | --- | --- | --- | --- | --- | --- | --- | --- | --- | --- | --- | --- | --- |
|  |  | ***prec*** | ***rec*** | ***F1*** | ***AUC*** | ***prec*** | ***rec*** | ***F1*** | ***AUC*** | ***prec*** | ***rec*** | ***F1*** | ***AUC*** |
| **DT** | ***NI*** | 0.92 | 0.87 | 0.90 | **0.73** | 0.99 | 0.99 | 0.99 | **0.68** | 0.98 | 1.00 | 0.99 | **0.71** |
|  | ***I*** | **0.57** | **0.72** | **0.64** |  | **0.42** | **0.66** | **0.58** |  | **0.74** | **0.56** | **0.64** |  |
| **RF** | ***NI*** | 0.93 | 0.91 | 0.92 | 0.75 | 0.99 | 1.00 | 0.99 | 0.70 | 0.99 | 0.99 | 0.99 | 0.73 |
|  | ***I*** | 0.71 | 0.63 | 0.70 |  | 0.38 | 0.72 | 0.60 |  | 0.78 | 0.58 | 0.66 |  |
| **LR** | ***NI*** | 0.83 | 0.77 | 0.80 | 0.71 | 0.98 | 0.99 | 0.99 | 0.61 | 0.98 | 0.98 | 0.98 | 0.63 |
|  | ***I*** | 0.68 | 0.64 | 0.65 |  | 0.58 | 0.33 | 0.42 |  | 0.73 | 0.48 | 0.55 |  |
