# Supplementary material for: Effective injury forecasting in soccer with GPS training data and machine learning
Source: PLoS One. 2018 Jul 25;13(7):e0201264. doi: 10.1371/journal.pone.0201264 (PMC6059460; doi:10.1371/journal.pone.0201264)
Supplement: S7 Table — Features extracted by RFECV in each Ti built as the season went by. (DOCX) [file pone.0201264.s016.docx]

| **T_i_** | **RFECV** |
| --- | --- |
| ***6*** | d_MET_^(EWMA)^, DEC_3_^(ACWR)^, PI^(EWMA)^ |
| ***7*** | PI^(EWMA)^, d_HSR_^(EWMA)^, d_TOT_^(MSWR)^ |
| ***8*** | PI^(EWMA)^, d_HSR_^(EWMA)^, d_TOT_^(MSWR)^ |
| ***9*** | d_HSR_^(EWMA)^, ACC_2_^(EWMA)^, d_HML/m_^(ACWR)^, d_EXP_^(MSWR)^, PI^(EWMA)^ |
| ***10*** | d_HSR_^(EWMA)^, PI^(EWMA)^ |
| ***11*** | DEC_2_, d_HSR_^(EWMA)^, d_HML/m_^(EWMA)^, d_EXP_^(MSWR)^, PI^(EWMA)^ |
| ***12*** | ACC_2_, d_HSR_^(EWMA)^, DEC_3_^(ACWR)^, PI^(EWMA)^ |
| ***13*** | d_HSR_^(EWMA)^, d_HSR_^(ACWR)^, PI^(EWMA)^, FI^(MSWR)^ |
| ***14*** | PI^(EWMA)^, d_HSR_^(EWMA)^, d_TOT_^(MSWR)^ |
| ***15*** | ACC_2_^(EWMA)^, PI^(EWMA)^ |
| ***16*** | PI^(EWMA)^, d_HSR_^(EWMA)^, d_TOT_^(MSWR)^ |
| ***17*** | PI^(EWMA)^, d_HSR_^(EWMA)^, d_TOT_^(MSWR)^ |
| ***18*** | PI^(EWMA)^, d_HSR_^(EWMA)^, d_TOT_^(MSWR)^ |
| ***19*** | PI^(EWMA)^, d_HSR_^(EWMA)^, d_TOT_^(MSWR)^ |
| ***20*** | PI^(EWMA)^, d_HSR_^(EWMA)^, d_TOT_^(MSWR)^ |
| ***21*** | PI^(EWMA)^, d_HSR_^(EWMA)^, d_TOT_^(MSWR)^ |
