# Supplementary figures and images for: Effective injury forecasting in soccer with GPS training data and machine learning
Source: PLoS One. 2018 Jul 25;13(7):e0201264. doi: 10.1371/journal.pone.0201264 (PMC6059460; doi:10.1371/journal.pone.0201264)

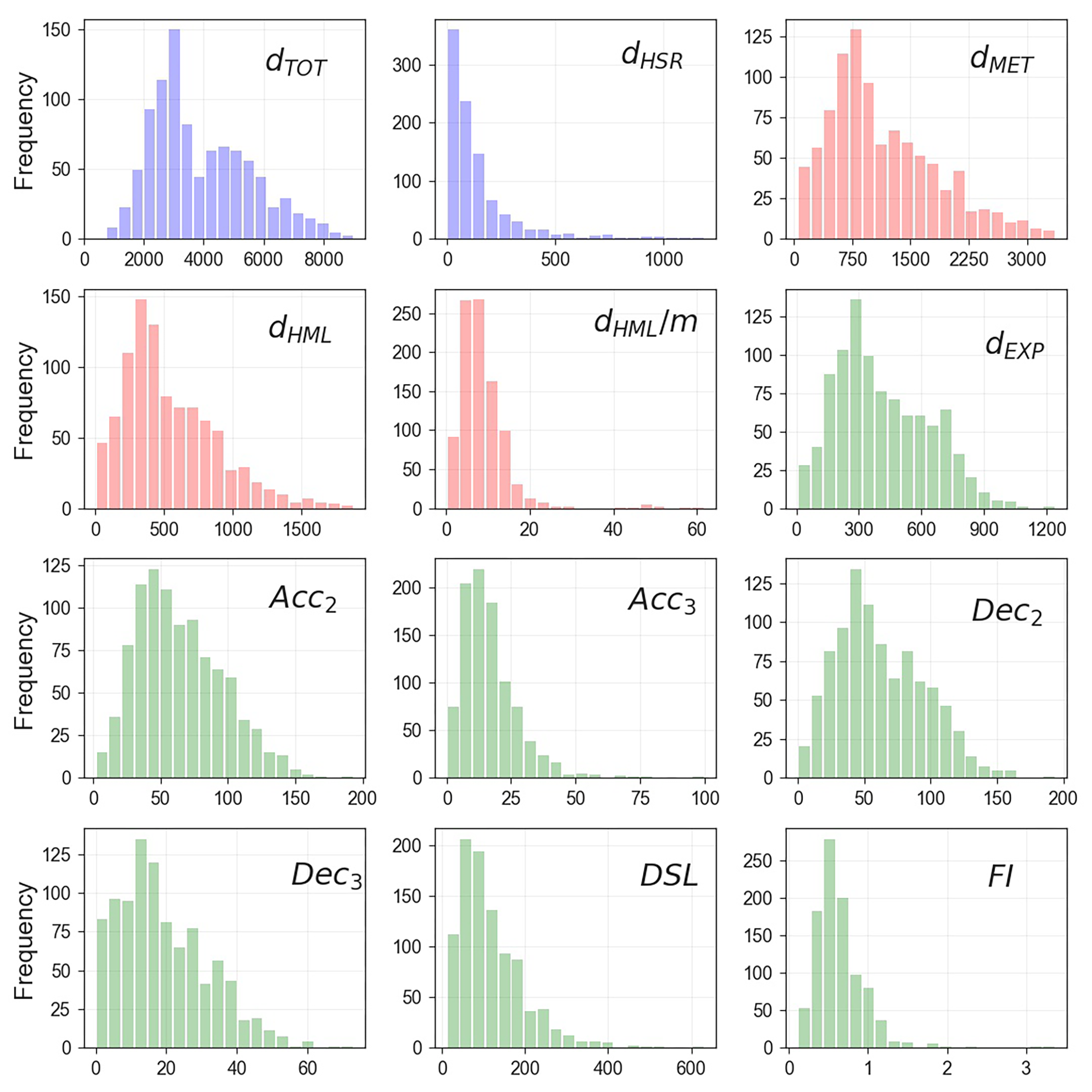

Supplement: S1 Fig — We provide three categories of training workload features: kinematic features (blue), metabolic features (red) and mechanical features (green). (TIF) [file pone.0201264.s017.tif]

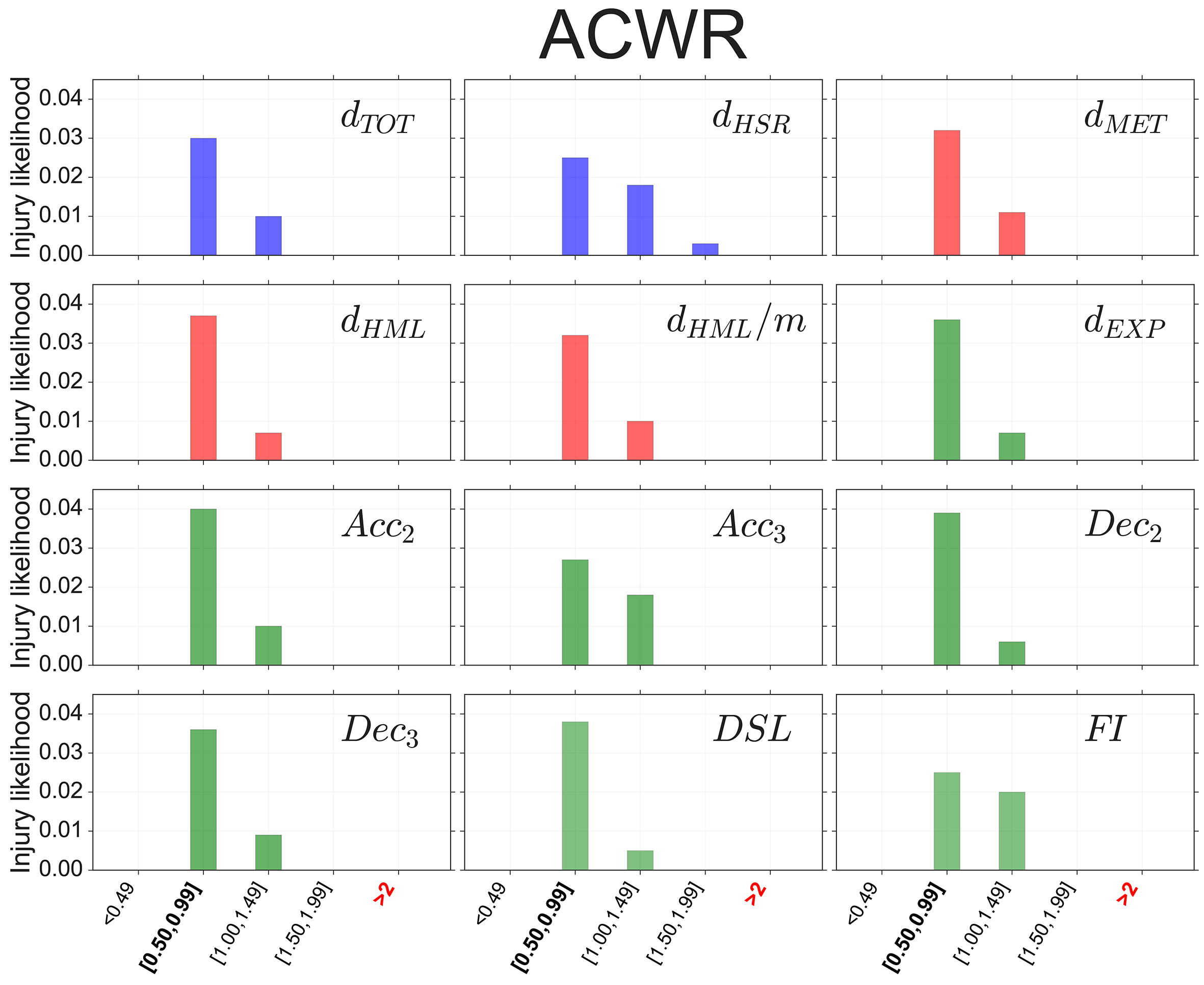

Supplement: S2 Fig — The plots show Injury Likelihood (IL) for pre- defined ACWR groups [29], for every of the 12 training workload features considered in our study. Bars are colored according to feature categorization defined in Table 1. (TIF) [file pone.0201264.s018.tif]

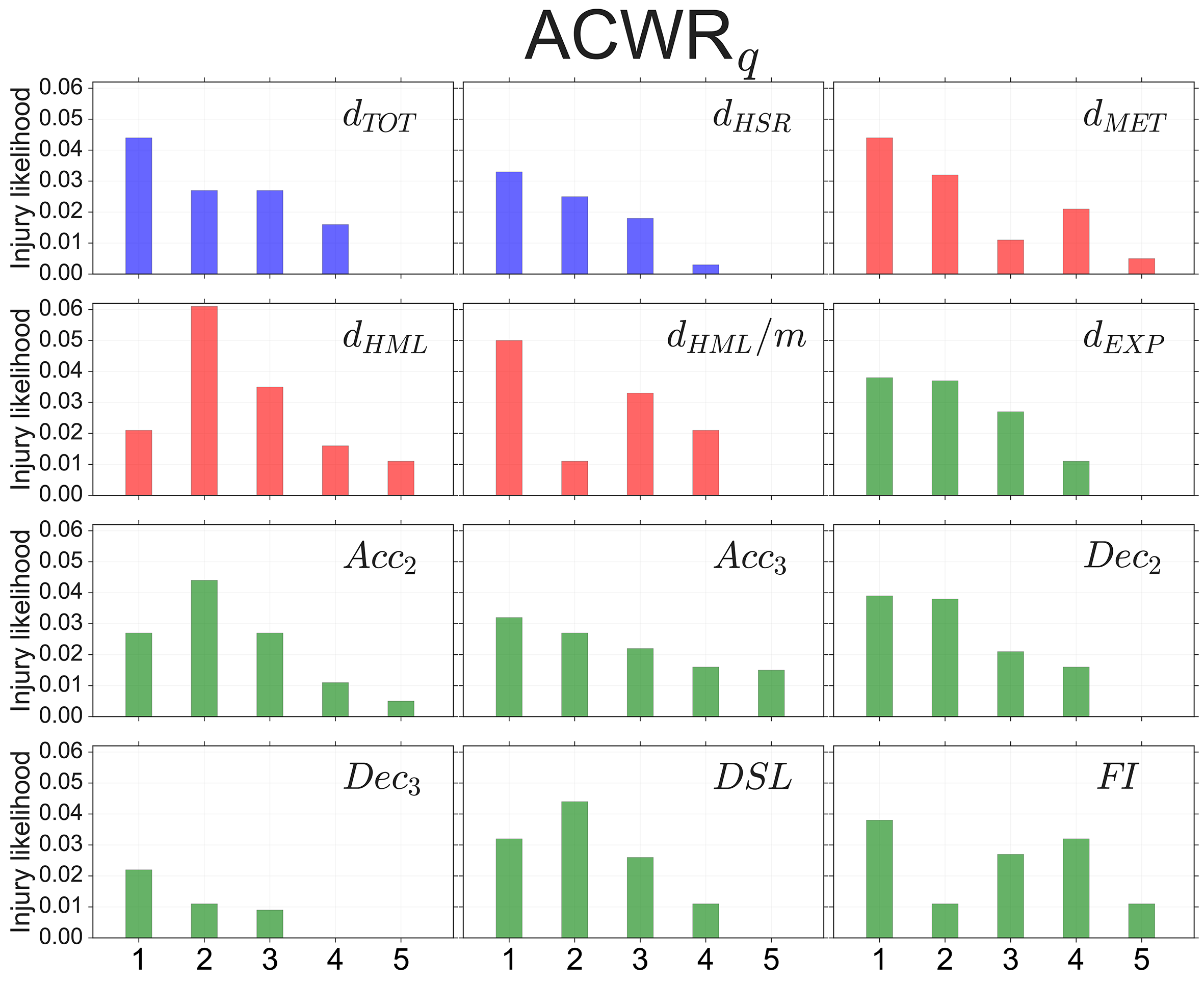

Supplement: S3 Fig — The plots show IL for the ACWR groups defined the quantiles of the distribution, for every of the 12 training workload features considered in our study. We provide three categories of training workload features: kinematic features (blue), metabolic features (red) and mechanical features (green). (TIF) [file pone.0201264.s019.tif]

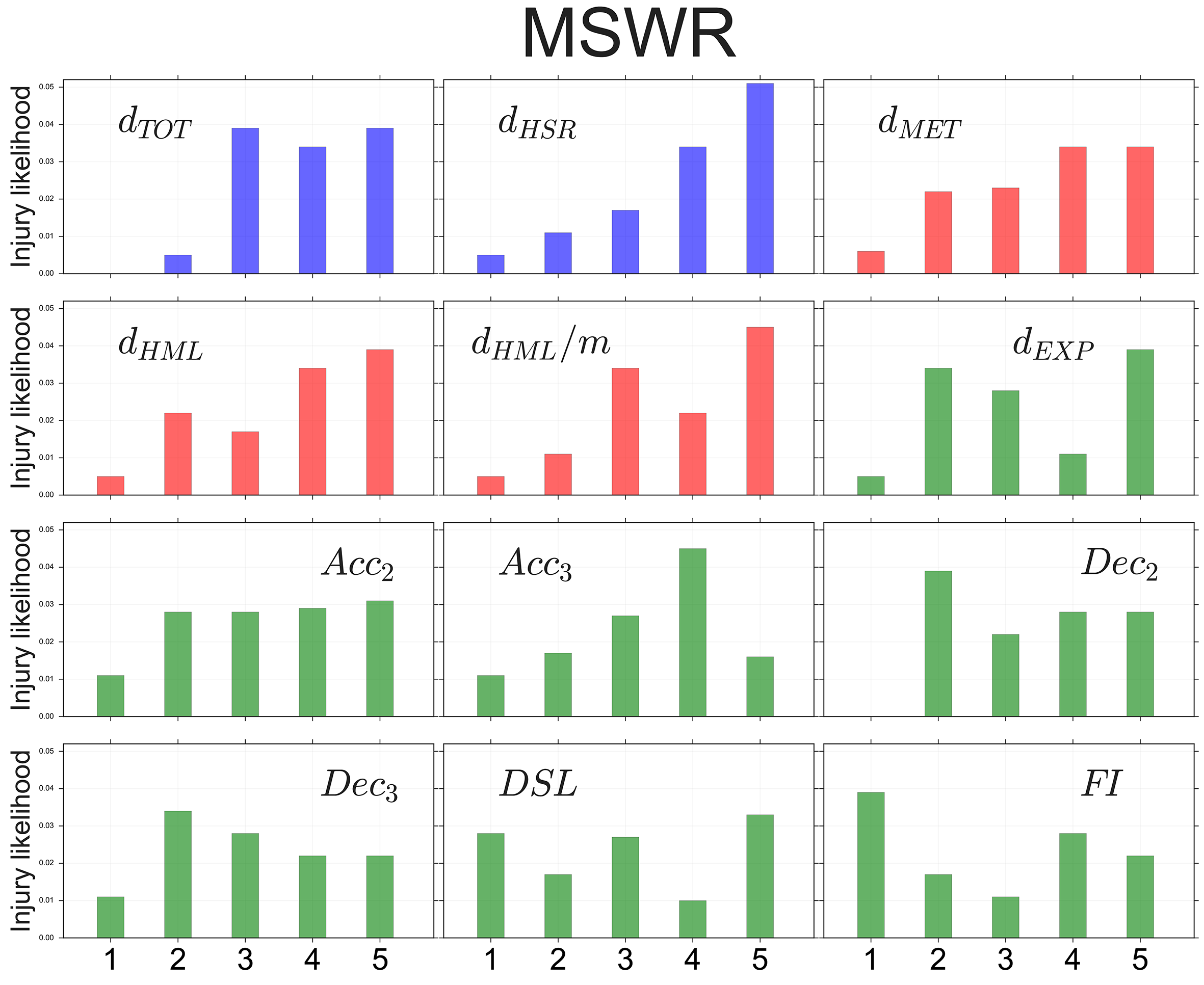

Supplement: S4 Fig — The plots show the Injury Likelihood (IL) for the MSWR groups for every of the 12 training workload features considered in our study. Bars are colored according to feature categorization defined in Table 1. (TIF) [file pone.0201264.s020.tif]

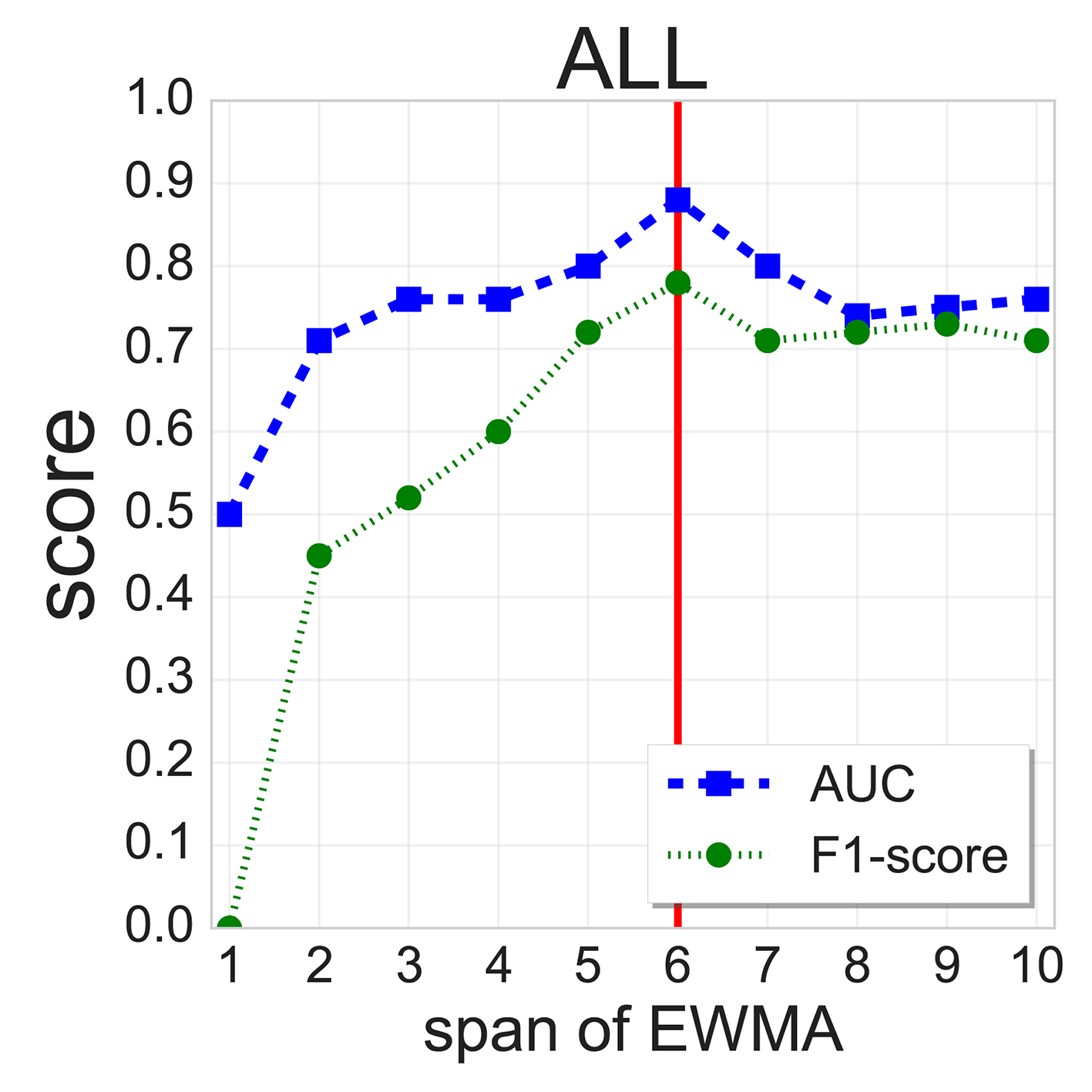

Supplement: S5 Fig — The red line reflects the best span to injury prediction. (TIF) [file pone.0201264.s021.tif]
